# Extracellular Matrix Remodeling in the Retina and Optic Nerve of a Novel Glaucoma Mouse Model

**DOI:** 10.3390/biology10030169

**Published:** 2021-02-24

**Authors:** Jacqueline Reinhard, Susanne Wiemann, Sebastian Hildebrandt, Andreas Faissner

**Affiliations:** Department of Cell Morphology and Molecular Neurobiology, Faculty of Biology and Biotechnology, Ruhr-University Bochum, Universitaetsstrasse 150, 44780 Bochum, Germany; susanne.wiemann@rub.de (S.W.); sebastian.hildebrandt@rub.de (S.H.); andreas.faissner@rub.de (A.F.)

**Keywords:** extracellular matrix, fibronectin, glaucoma, glycoproteins, intraocular pressure, laminins, optic nerve, proteoglycans, retina, tenascins

## Abstract

**Simple Summary:**

Glaucoma is a leading cause of blindness worldwide, and increased age and intraocular pressure (IOP) are the major risk factors. Glaucoma is characterized by the death of nerve cells and the loss of optic nerve fibers. Recently, evidence has accumulated indicating that proteins in the environment of nerve cells, called the extracellular matrix (ECM), play an important role in glaucomatous neurodegeneration. Depending on its constitution, the ECM can influence either the survival or the death of nerve cells. Thus, the aim of our study was to comparatively explore alterations of various ECM molecules in the retina and optic nerve of aged control and glaucomatous mice with chronic IOP elevation. Interestingly, we observed elevated levels of blood vessel and glial cell-associated ECM components in the glaucomatous retina and optic nerve, which could be responsible for various pathological processes. A better understanding of the underlying signaling mechanisms may help to develop new diagnostic and therapeutic strategies for glaucoma patients.

**Abstract:**

Glaucoma is a neurodegenerative disease that is characterized by the loss of retinal ganglion cells (RGC) and optic nerve fibers. Increased age and intraocular pressure (IOP) elevation are the main risk factors for developing glaucoma. Mice that are heterozygous (HET) for the mega-karyocyte protein tyrosine phosphatase 2 (PTP-Meg2) show chronic and progressive IOP elevation, severe RGCs loss, and optic nerve damage, and represent a valuable model for IOP-dependent primary open-angle glaucoma (POAG). Previously, evidence accumulated suggesting that glaucomatous neurodegeneration is associated with the extensive remodeling of extracellular matrix (ECM) molecules. Unfortunately, little is known about the exact ECM changes in the glaucomatous retina and optic nerve. Hence, the goal of the present study was to comparatively explore ECM alterations in glaucomatous PTP-Meg2 HET and control wild type (WT) mice. Due to their potential relevance in glaucomatous neurodegeneration, we specifically analyzed the expression pattern of the ECM glycoproteins fibronectin, laminin, tenascin-C, and tenascin-R as well as the proteoglycans aggrecan, brevican, and members of the receptor protein tyrosine phosphatase beta/zeta (RPTPβ/ζ) family. The analyses were carried out in the retina and optic nerve of glaucomatous PTP-Meg2 HET and WT mice using quantitative real-time PCR (RT-qPCR), immunohistochemistry, and Western blot. Interestingly, we observed increased fibronectin and laminin levels in the glaucomatous HET retina and optic nerve compared to the WT group. RT-qPCR analyses of the laminins α4, β2 and γ3 showed an altered isoform-specific regulation in the HET retina and optic nerve. In addition, an upregulation of tenascin-C and its interaction partner RPTPβ/ζ/phosphacan was found in glaucomatous tissue. However, comparable protein and mRNA levels for tenascin-R as well as aggrecan and brevican were observed in both groups. Overall, our study showed a remodeling of various ECM components in the glaucomatous retina and optic nerve of PTP-Meg2 HET mice. This dysregulation could be responsible for pathological processes such as neovascularization, inflammation, and reactive gliosis in glaucomatous neurodegeneration.

## 1. Introduction

Globally, glaucoma is the second leading cause of severe visual impairment and irreversible blindness [1]. Its prevalence is steadily increasing, and it is expected that the number of people affected will rise from around 76 million in 2020 to around 112 million in 2040 [2]. Glaucoma comprises a group of chronic, progressive opticus neuropathies characterized by changes in the optic nerve head, degeneration of retinal ganglion cells (RGCs) and optic nerve fibers, and visual field loss [1]. Although glaucoma is a complex and multifactorial disease, the most important risk factors are an increased age and a high intraocular pressure (IOP).

Mice that are heterozygous (HET) for the megakaryocyte protein tyrosine phosphatase 2 (PTP-Meg2) represent an excellent model to investigate the underlying pathomechanism of IOP-induced primary open-angle glaucoma (POAG) [3]. PTP-Meg2 HET mice show a progressive IOP increase upon 10 weeks of age, which reaches its peak at 28 weeks of age. The glaucomatous phenotype of HET mice is characterized by the degeneration of RGCs and optic nerve fibers. In addition, neurodegenerative processes in this glaucoma mouse model are accompanied with deficits of retinal function and reactive micro- and macrogliosis.

In recent years, evidence has accumulated suggesting that glaucomatous damage is associated with an extensive remodeling of various extracellular matrix (ECM) molecules. Little is known, however, about the exact ECM changes in the glaucomatous retina and optic nerve. The ECM is a complex network of different macromolecules in the intercellular space that provide structural and mechanical support and can be divided into glycoproteins and proteoglycans. Components of the ECM are involved in various cellular events by binding to cell surface receptors, thereby regulating proliferation, migration, differentiation, and survival [4,5,6,7,8]. Furthermore, they are the main components of glial scars and provide an inhibitory environment for the regeneration and migration of neurons [9].

A characteristic of glaucoma is a morphological change in the optic nerve head [10,11]. In POAG, the increased IOP leads to a deformation of the optic nerve head, which is rich in ECM molecules. Previous studies suggest that changes in the composition of ECM components, including collagen, elastin, and tenascin-C, promote axonal damage [12,13,14,15]. In addition, a remodeling of various ECM molecules is associated with neurodegeneration of the retina and optic nerve after ischemia [16].

In the present study, we analyzed ECM remodeling in the retina and optic nerve of the transgenic IOP-dependent PTP-Meg2 glaucoma mouse model. We have specifically characterized the expression pattern of the glycoproteins fibronectin, laminin, tenascin-C, and tenascin-R as well as the chondroitin sulfate proteoglycans (CSPGs) aggrecan, brevican, and receptor protein tyrosine phosphatase beta/zeta (RPTPβ/ζ)/phosphacan.

Fibronectin is a structural basement membrane component of retinal vessels and the retinal pigment epithelium [17]. An increased fibronectin staining in the retinal vasculature and inner limiting membrane (ILM) was described in a diabetic retinopathy animal model [18]. In glaucoma, the accumulation of fibronectin leads to a dysfunction of the trabecular meshwork and IOP elevation [19,20]. It was recently reported that the extra domain A of fibronectin and the Toll-like receptor 4 (TLR4) contribute to transforming growth factor beta 2 induced ocular hypertension [21].

Members of the laminin family are major components of the retinal vascular basement membrane and play a functional role in differentiation and maintenance [22,23]. The laminin isoform α4 is expressed by endothelial cells of basement membranes and is involved in the growth of microvessels [24,25]. Immunoreactivity of the laminin α4 chain was observed in the ganglion cell layer (GCL) and in close association with Müller glia endfeet [22]. The β2 and γ3 laminin chains are crucial for the formation and stability of the ILM and influence the differentiation and migration of retinal astrocytes [26,27].

In the retina, the glycoprotein tenascin-C is expressed by horizontal and amacrine cells, and mainly associates with the plexiform layers. Astrocytes express huge amounts of tenascin-C in the optic nerve [9,28,29]. Tenascin-C is upregulated under pathological conditions and involved in neuroinflammatory and glial reactions [30,31,32]. Upregulation of tenascin-C has been described in various eye diseases, such as diabetic retinopathy and glaucoma [28]. Additionally, an accumulation of tenascin-C was observed in an IOP-dependent and IOP-independent autoimmune glaucoma animal model [33,34].

Tenascin-R can influence neurite outgrowth as well as neural and glial adhesion [35,36,37]. In the retina, tenascin-R is released by horizontal cells and accumulates in the plexiform layers. Oligodendrocytes express huge amounts of tenascin-R in the optic nerve. Here, tenascin-R is closely associated with myelinated optic nerve fibers and nodes of Ranvier [38,39]. Both tenascin-C and -R can interact with various ECM molecules, for example with fibronectin, lecticans and members of the RPTPβ/ζ family [40].

CSPGs are highly concentrated in the glial scar and limit the ability for axonal regeneration [41,42,43]. The lecticans aggrecan and brevican are released by reactive astrocytes. Both lecticans are located in the plexiform layers of the retina, while a widespread extracellular staining pattern can be observed in the optic nerve [16,44].

Different splice variants of RPTPβ/ζ have been described to interact with several adhesion molecules and tenascins. The receptor isoforms RPTPβ/ζ_long_ and RPTPβ/ζ_short_ are expressed by glial cells and the expression of the secreted isoform RPTPβ/ζ/phosphacan is restricted to Müller glia in the adult retina [9,45]. An increased RPTPβ/ζ/phosphacan expression in the retina and optic nerve of an IOP-independent autoimmune glaucoma rat model was previously shown [16,33].

The aim of our study was to investigate the remodeling of the ECM glycoproteins fibronectin, laminin, tenascin-C, and tenascin-R as well as the CSPGs aggrecan, brevican, and RPTPβ/ζ/phosphacan in the retina and optic nerve after chronic IOP elevation. Therefore, we comparatively explore the expression pattern of these ECM components in aged wild type (WT) and PTP-Meg2 HET glaucoma mice by quantitative real-time PCR (RT-qPCR), immunohistochemistry, and Western blot analyses.

## 2. Materials and Methods

### 2.1. Animals

Male and female WT and glaucomatous PTP-Meg2 HET mouse littermates were used at the age of 28 weeks [3]. Mice were held in the animal facility of the Ruhr-University Bochum and kept in a 12 h–12 h light–dark cycle with free access to food and water.

### 2.2. RNA Isolation, cDNA Synthesis, and RT-qPCR

Retinae and optic nerves were dissected and stored at −80 °C until purification (*n* = 5/group). The isolation of the total RNA was performed according to the manufacturer‘s protocol using the Gene Elute Mammalian Total RNA Miniprep Kit (Sigma–Aldrich, St. Louis, MO, USA). The RNA concentration was measured photometrically using the BioSpectrometer^®^ (Eppendorf, Hamburg, Germany). For cDNA synthesis, 1 μg RNA was reverse-transcribed with random hexamer primers using a cDNA synthesis kit (Thermo Fisher Scientific, Waltham, MA, USA).

The Light Cycler 96^®^ System and SYBR Green I (Roche Applied Science, Mannheim, Germany) was used for RT-qPCR analyses as previously described [3]. Primer pairs were designed using the ProbeFinder Assay Design Software (Roche Diagnostics, Mannheim, Germany; Table 1). Primer efficiencies were calculated by a dilution series of 5, 25, and 125 ng cDNA. ECM expression in the retina and optic nerve was normalized by using primer pairs for the housekeeping genes *β-actin* (*Actb*) and *cyclophilin D* (*Ppid*), respectively.

### 2.3. Immunohistochemistry and Confocal Laser Scanning Microscopy

For immunohistochemistry, eyes and optic nerves were fixed in 4% paraformaldehyde and dehydrated in 30% sucrose in 1× phosphate-buffered saline (1× PBS) and embedded in Tissue-Tek freezing medium (Thermo Fisher Scientific, Cheshire, UK). Retinal cross-sections and longitudinal optic nerve sections were cut with a thickness of 16 μm at a cryostat (CM3050 S, Leica, Nussloch, Germany). Sections were incubated in blocking solution (1% bovine serum albumin, Sigma-Aldrich, 3% goat serum, Dianova, Hamburg, Germany, and 0.5% Triton^TM^-X-100, Sigma-Aldrich in 1× PBS) for 1 h at room temperature (RT). The sections were then incubated with primary antibodies (Table 2) in blocking solution overnight. After washing with 1× PBS, the sections were incubated for 2 h with secondary antibody (Table 2) in blocking solution without Triton^TM^-X-100. All stainings included a secondary, antibody only, negative control. Cell nuclei were stained with TO-PRO-3 (1:400; Thermo Fisher Scientific). Two sections from each animal (*n* = 5/group) were analyzed at a 400× magnification with a confocal laser scanning microscope (LSM 510 META, Zeiss, Göttingen, Germany). Two peripheral and two central images per retinal section and three images from the proximal to the distal part of the myelinated optic nerve (200× magnification) were taken. Laser lines and emission filters were set using the Zeiss ZEN black software (Zeiss). A fixed cutting window was defined with Coral Paint Shop Pro X8 (Coral Corporation, CA, USA). Measurements of the stained signal area [%]/image were done using ImageJ (ImageJ 1.51 w, National Institutes of Health; Bethesda, MD, USA) as previously described [16,33]. For analyses, we determined the background subtraction, the lower threshold and the upper threshold (Table 3).

### 2.4. Western Blotting

WT and HET retinal tissue (*n* = 4–6/group) was homogenized in 100 µL lysis buffer (60 mM n-octyl-β-D-glucopyranoside, 50 mM sodium acetate, 50 mM Tris chloride, pH 8.0), which contains a protease inhibitor cocktail (Sigma–Aldrich) on ice for 1 h. Afterwards, the samples were centrifuged at 4 °C for 30 min and the supernatant was used to determine the protein concentration using the BCA Protein Assay Kit (Pierce, Thermo Fisher Scientific, Rockford, IL, USA). 4× sodium dodecyl sulfate (SDS) buffer was given to the samples (20–40 µg), which were then denaturized at 94 °C for 5 min and separated by electrophoresis using 4–10% polyacrylamide gradient gels. By Western blotting (1.5 h and 75 mA) proteins were transferred to a polyvinylidene difluoride membrane (Roth, Karlsruhe, Germany). Subsequently, membranes were blocked in blocking solution (5% *w*/*v* milk powder in Tris-buffered saline (TBS) with 0.05% Tween 20, TBST) for 1 h at RT. Then membranes were incubated with primary antibodies (Table 4) diluted in blocking solution at 4 °C overnight. After washing in TBST, horseradish peroxidase (HRP)-coupled secondary antibodies (Table 4) diluted in blocking solution were applied and incubated at RT for 1 h. The membranes were then washed in TBST and TBS for 10–15 min each and incubated for 5 min in Enhanced Chemiluminescence Substrate solution (1:1 mixed; Bio-Rad Laboratories GmbH, München, Germany). Protein bands were detected with a MicroChemi Chemiluminescence Reader (Biostep, Burkhardtsdorf, Germany). Band intensities were measured using ImageJ software and normalized to the reference protein α-Tubulin. The normalized Western blot values are shown as arbitrary units (a.u.).

### 2.5. Statistics

The stained signal area [%]/image in retinal and optic nerve sections as well as normalized protein band intensity of both genotypes were analyzed with the Student‘s *t*-test (V12; StatSoft Europe, Hamburg, Germany). The mean ± standard deviation (SD) is given in the result section. Data are presented as box plots showing mean ± standard error mean (SEM) ± SD. Cq-values of RT-qPCR analyses were evaluated with the Light Cycler 96^®^ Software (V1.1; Roche Applied Science) and analyzed with the software REST^©^ (Relative expression software tool 2009; QIAGEN GmbH, Hilden, Germany) using a pairwise fixed reallocation and randomization test [51]. Data are presented as box plots showing median ± quartile ± minimum/maximum. *p*-values below 0.05 were considered statistically significant.

## 3. Results

### 3.1. Remodeling of Glycoproteins in the Glaucomatous Retina of PTP-Meg2 HET Mice

**Figure 1:**
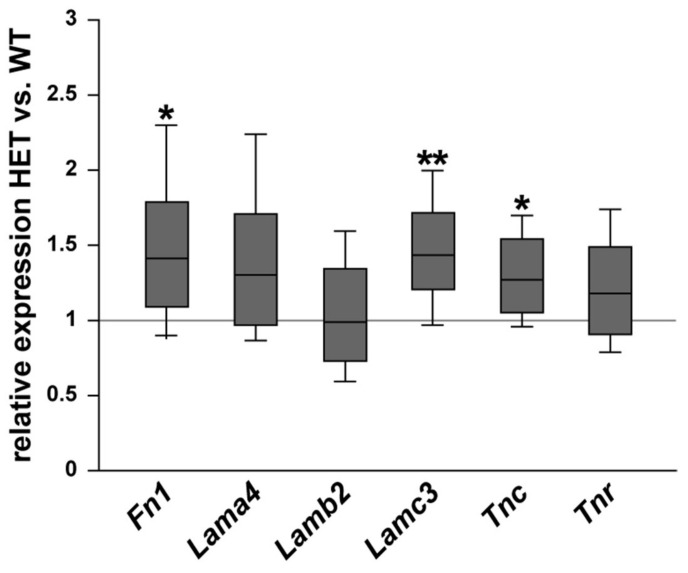
Increased *Fn1*, *Lamc3,* and *Tnc* mRNA expression levels in the glaucomatous retina of HET mice. Relative expression of ECM glycoproteins in WT and HET retinae was determined by RT-qPCR. Our results showed a significantly increased *Fn1*, *Lamc3* and *Tnc* expression in the HET retina. Both groups showed a comparable *Lama4*, *Lamb2,* and *Tnr* expression. Groups were compared using a pairwise fixed reallocation and randomization test. Data are shown as median ± quartile ± minimum/maximum. * *p* < 0.05, ** *p* < 0.01. *n* = 5/group.

First, we examined the mRNA expression of various ECM glycoproteins in the retina of WT and glaucomatous HET mice. The expression of *fibronectin* (*Fn1*), the laminin isoforms *α4* (*Lama4*), *β2* (*Lamb2*), and *γ3* (*Lamc3*) as well as tenascin-C (*Tnc*) and tenascin-R (*Tnr*) were analyzed via RT-qPCR (Figure 1). *Fn1* (1.44-fold; *p* = 0.03) and *Tnc* (1.27-fold; *p* = 0.04) were significantly upregulated in the HET compared to the WT retina. The *Tnr* mRNA level was comparable in both groups (1.18-fold; *p* = 0.20). *Lamc3* (1.48-fold; *p* = 0.002) was significantly upregulated in the HET group, while *Lama4* (1.34-fold; *p* = 0.08) and *Lamb2* (0.99-fold; *p* = 0.94) expression levels were comparable in the WT and HET group.

**Figure 2:**
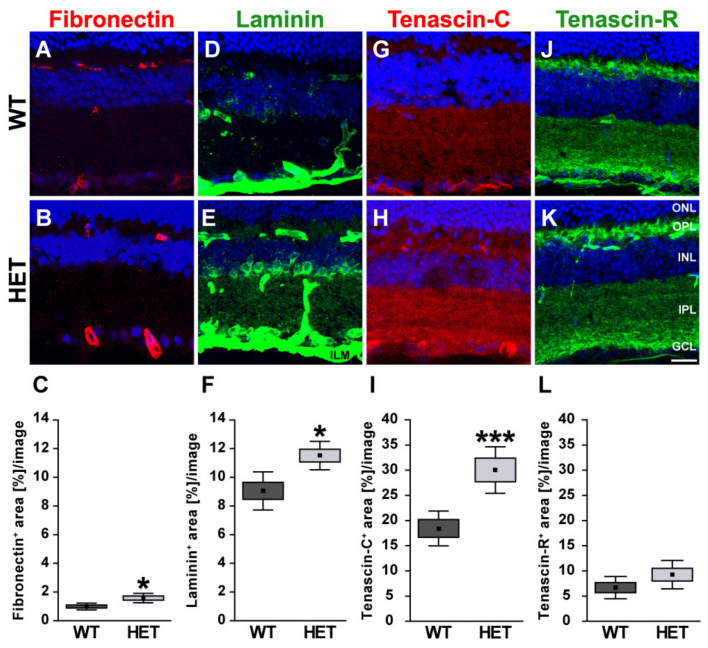
Enhanced immunoreactivity of ECM glycoproteins in the glaucomatous HET retina. Most representative immunohistochemical ECM stainings are shown. Fibronectin (red; (**A**,**B**)) and laminin (green; (**D**,**E**)) signals were found in blood vessels. A prominent laminin immunoreactivity was also found in the INL, GCL, and ILM. Staining of tenascin-C (red; (**G**,**H**)) and tenascin-R (green; (**J**,**K**)) was localized to the plexiform layers. The immunoreactivity of the glycoproteins fibro-nectin, laminin and tenascin-C was significantly upregulated in HET retinae (**C**,**F**,**I**). A comparable tenascin-R signal could be detected in HET and WT retinae (**L**). Cell nuclei were labeled with TO-PRO-3 (blue). Groups were analyzed by Student‘s *t*-test. Data are shown as mean ± SEM ± SD. * *p* < 0.05, *** *p* < 0.001. *n* = 5/group. Scale bar = 20 μm. Abbreviations: ONL—outer nuclear layer, OPL—outer plexiform layer, INL—inner nuclear layer, IPL—inner plexiform layer, GCL—ganglion cell layer, ILM—inner limiting membrane.

**Figure 3:**
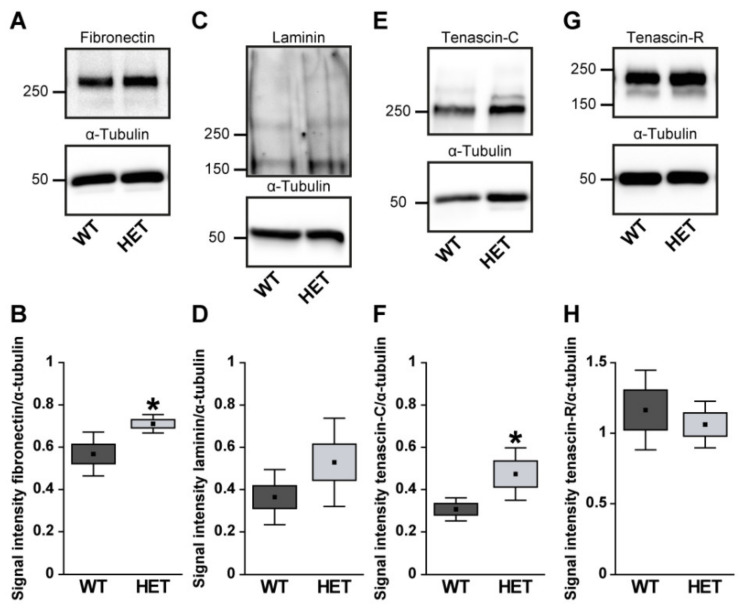
Western blot analyses of glycoproteins of WT and HET retinal tissue. A significantly increased protein band intensity was observed for fibronectin (**A**,**B**) and tenascin-C (**E**,**F**) in glaucomatous HET mice. Relative protein quantification revealed a slightly enhanced band intensity of laminin (**C**,**D**) in the HET group. Comparable protein levels of tenascin-R were found in both genotypes (**G**,**H**). Groups were analyzed by Student‘s *t*-test. Data are shown as mean ± SEM ± SD. * *p* < 0.05. *n* = 4–6/group.

Next, retinal cross-sections were immunohistochemically stained for the glycoproteins. The fibronectin staining pattern was clearly associated with blood vessels (Figure 2A,B). Compared to WT retinae, HET retinae showed a significantly increased fibronectin immunoreactivity (HET: 1.58 ± 0.32 area [%]/image vs. WT: 0.99 ± 0.23 area [%]/image; *p* = 0.01; Figure 2C). In line with these findings, quantitative Western blot analysis also revealed a significantly increased intensity of fibronectin protein levels in the HET condition (HET: 0.71 ± 0.10 a.u. vs. WT: 0.57 ± 0.04 a.u.; *p* = 0.02; Figure 3A,B). The signal of laminin was mainly localized to the ILM and retinal blood vessels. In addition, laminin-immunoreactive cells were seen in the inner nuclear layer (INL; Figure 2D,E). A significantly increased laminin^+^ area was detected in the HET retina (11.45 ± 0.97 area [%]/image) compared to the WT retina (9.00 ± 1.28 area [%]/image; *p* = 0.01; Figure 2F). Western blot analyses revealed that the laminin protein level tended to be increased in the HET group, but this was not significant (HET: 0.52 ± 0.21 a.u. vs. WT: 0.36 ± 0.13 a.u.; *p* = 0.13; Figure 3C,D).

In the retina, displaced amacrine, amacrine and horizontal cells are the cellular source of Tnc expression [29]. Tenascin-C immunoreactivity of retinal cross-sections was mainly observed in the plexiform layers and in the GCL (Figure 2G,H). The quantification revealed a significant upregulation of the tenascin-C^+^ area in the HET (29.97 ± 12.87 area [%]/image) compared to WT retinae (18.40 ± 9.31 area [%]/image; *p* < 0.001; Figure 2I). Western blots also showed enhanced tenascin-C protein levels in the glaucomatous HET retina (HET: 0.48 ± 0.12 a.u. vs. WT: 0.30 ± 0.07 a.u.; *p* = 0.04; Figure 3E,F).

Tenascin-R^+^ staining was found in the outer and inner plexiform layer (OPL and IPL), and GCL. However, no differences in the retinal staining area could be detected in both genotypes (HET: 9.26 ± 2.82 area [%]/image vs. WT: 6.69 ± 2.22 area [%]/image; *p* = 0.15; Figure 2J–L). Western blot analyses verified comparable protein levels of tenascin-R in the retina of both genotypes (WT: 1.16 ± 0.28 a.u. vs. HET: 1.06 ± 0.16 a.u.; *p* = 0.55; Figure 3G,H).

### 3.2. Remodeling of Glycoproteins in the Glaucomatous Optic Nerve of PTP-Meg2 HET Mice

**Figure 4:**
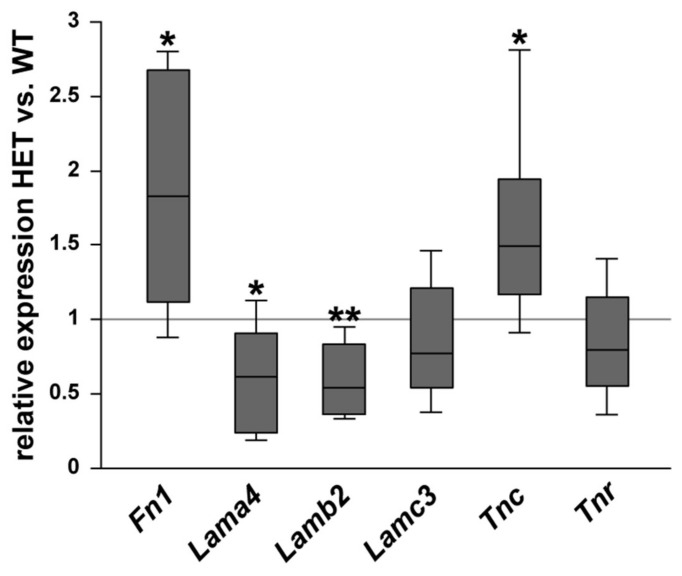
Diminished glycoprotein expression in the glaucomatous optic nerve of HET animals. Comparison of ECM glycoprotein expression in optic nerves of WT and HET mice was revealed by RT-qPCR analyses. Significantly higher *Fn1* and *Tnc* expression levels were observed in the optic nerves of HET compared to WT mice. Comparable mRNA levels were found for *Tnr* and *Lamc3*. In contrast, the expression of *Lama4* and *Lamb2* was significantly downregulated in the optic nerves of HET compared to WT mice. Groups were compared using a pairwise fixed reallocation and randomization test. Data are shown as median ± quartile ± minimum/maximum. * *p* < 0.05, ** *p* < 0.01. *n* = 5/group.

Then, the relative mRNA expression of the glycoproteins was determined in the optic nerve of WT and HET mice (Figure 4). A significant increase in *Fn1* (1.83-fold; *p* = 0.014) and *Tnc* (1.49-fold; *p* = 0.029) expression was observed in the HET group. No changes were found for *Lamc3* (0.77-fold; *p* = 0.22) and *Tnr* (0.80-fold; *p* = 0.23). Interestingly, the expression level of the laminin isoforms *Lama4* (0.62-fold; *p* = 0.026) and *Lamb2* (0.54-fold; *p* = 0.001) was significantly lower in the glaucomatous optic nerve.

**Figure 5:**
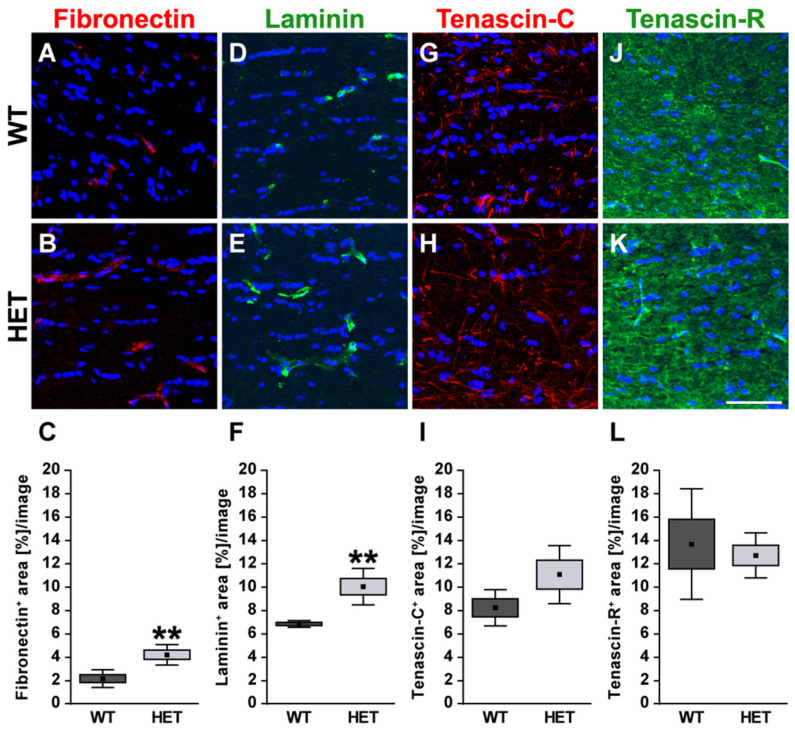
Enhanced staining signals of blood vessel-associated fibronectin and laminin in the glaucomatous optic nerve of HET mice. Most representative immunohistochemical ECM stainings are shown. Staining of the glycoproteins fibronectin (red; (**A**–**C**)) and laminin (green; (**D**–**F**)) was significantly increased in the HET optic nerves. Signals were limited to blood vessels. A thread-like-staining pattern was found for tenascin-C (red; (**G**,**H**)), while a widely extracellular staining pattern was observed for tenascin-R (green; (**J**,**K**)). Both tenascins showed a comparable staining area in the WT and HET group (**I**,**L**). Groups were analyzed by Student‘s *t*-test. Data are shown as mean ± SEM ± SD. ** *p* < 0.01. *n* = 5/group. Scale bar = 50 μm.

In the optic nerve, the immunoreactivity of the glycoproteins fibronectin and laminin was restricted to blood vessels (Figure 5A,B,D,E). HET mice (4.22 ± 0.88 area [%]/image) showed a significant increase in the fibronectin^+^ area compared to the WT (2.17 ± 0.76 area [%]/image; *p* = 0.004; Figure 5C). The statistical analysis also revealed a higher laminin^+^ area in the HET group (HET: 10.00 ± 1.57 area [%]/image vs. WT: 6.79 ± 0.28 area [%]/image; *p* = 0.002; Figure 5F). For tenascin-C, a thread-like staining pattern could be observed in optic nerve sections. In the optic nerve, astrocytes are the cellular source of tenascin-C [9,28,29]. The evaluation of the tenascin-C^+^ area showed a slightly enhanced immunopositive area in HET mice (WT: 8.21 ± 1.55 area [%]/image vs. HET: 11.04 ± 2.49 area [%]/image; *p* = 0.10; Figure 5G–I). Tenascin-R displayed a widely extracellular staining pattern in the optic nerve. Here, a comparable tenascin-R^+^ area was found in the WT (WT: 13.96 ± 3.73 area [%]/image) and HET (HET: 12.32 ± 8.04 area [%]/image; *p* = 0.69; Figure 5J–L) group.

### 3.3. Remodeling of Proteoglycans in the Glaucomatous Retina of PTP-Meg2 HET Mice

**Figure 6:**
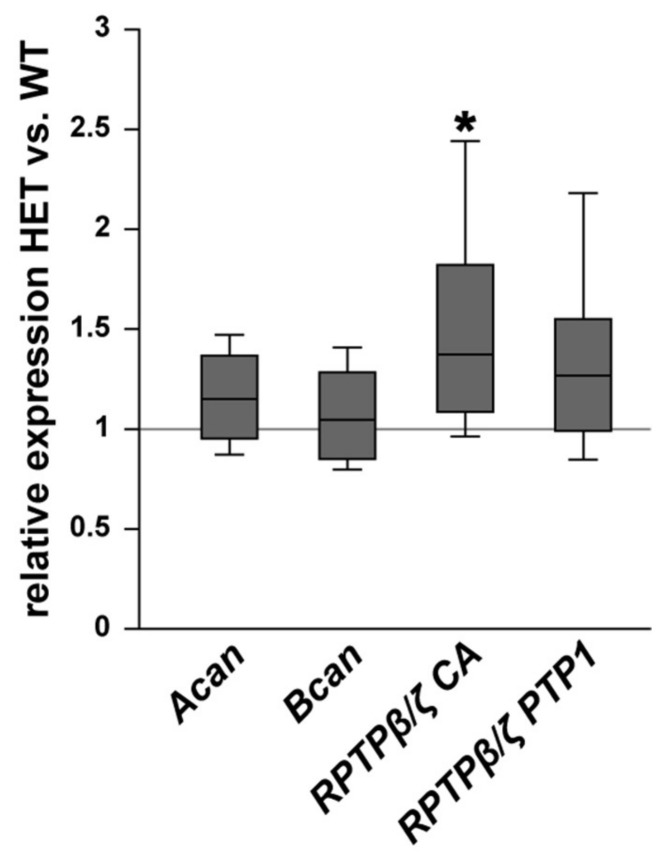
Upregulation of phosphacan expression in the glaucomatous HET retina. Analyses of *Acan*, *Bcan,* and *RPTPβ/ζ/phosphacan* mRNA expression in the retina of WT and HET mice by RT-qPCR. We observed a significant upregulation of *RPTPβ/ζ*/phosphacan detected by the primer pairs *RPTPβ/ζ* CA (all isoforms). However, no changes in the expression of the *RPTPβ/ζ* receptor variants, namely *RPTPβ/ζ_long_* and *RPTPβ/ζ_short_*, detected by the primer *RPTPβ/ζ* PTP1 (receptor isoforms RPTPβ/ζ_long_ and RPTPβ/ζ_short_) were found. Collectively, these findings indicate that phosphacan is upregulated in the HET retina. A comparable mRNA expression level was detected for *Acan* and *Bcan*. Groups were compared using a pairwise fixed reallocation and randomization test. Data are shown as median ± quartile ± minimum/maximum. * *p* < 0.05. *n* = 5/group.

In addition, the expression pattern of the proteoglycans aggrecan, brevican, and different RPTPβ/ζ isoforms was investigated in WT and HET retinae (Figure 6). The expression of *aggrecan* (*Acan)* was comparable in both genotypes (1.16-fold; *p* = 0.103). Comparable mRNA levels were also observed for *brevican* (*Bcan*; 1.05-fold; *p* = 0.61). The expression of all three *RPTPβ/ζ* isoforms, namely *RPTPβ/ζ_long_, RPTPβ/ζ_short_*, and phosphacan, was analyzed by the primer pairs *RPTPβ/ζ* CA, which were directed against the carbonic anhydrase-like (CA) domain. An increased expression level was found in the retinae of HET compared to the WT (1.4-fold; *p* = 0.013). For the receptor-type isoforms RPTPβ/ζ_long_ and RPTPβ/ζ_short_, which both have the PTP1 (protein tyrosine phosphatase domain 1), detected by the primer pairs RPTPβ/ζ PTP1, comparable levels were found in the HET condition (1.3-fold; *p* = 0.10). Based on both RPTPβ/ζ primer analyses, our results suggest that phosphacan is specifically upregulated in the HET retina, while the receptor-type isoforms appear to be constantly expressed in both groups.

**Figure 7:**
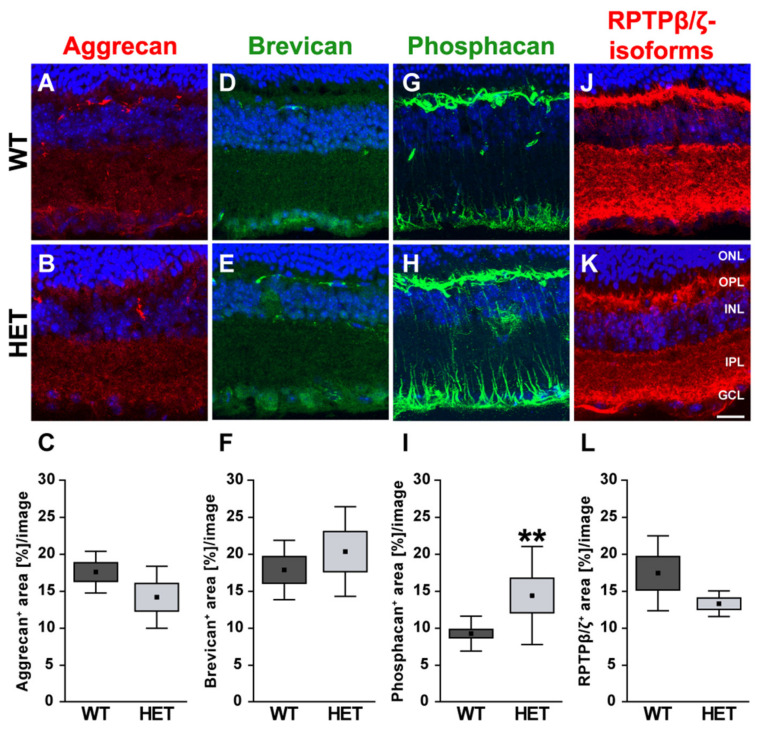
Enhanced phosphacan immunoreactivity in the glaucomatous retina of HET mice. Most representative immunohistochemical ECM stainings are shown. Stainings revealed a prominent signal for aggrecan (red; (**A**,**B**)), brevican (green; (**D**,**E**)) and all three RPTPβ/ζ isoforms (red; (**J**,**K**)) in the plexiform layers and the GCL. The phosphacan signal was restricted to Müller glia fibers (green; (**G**,**H**)). Quantification showed a significant upregulation of phosphacan in the HET retina (**I**). No changes were noted for aggrecan, brevican and all RPTPβ/ζ isoforms (**C**,**F**,**L**). TO-PRO-3 (blue) was used to detect the cell nuclei. Groups were analyzed by Student‘s *t*-test. Data are shown as mean ± SEM ± SD. ** *p* < 0.01. *n* = 5/group. Scale bar = 20 μm. Abbreviations: ONL—outer nuclear layer, OPL—outer plexiform layer, INL—inner nuclear layer, IPL—inner plexiform layer, GCL—ganglion cell layer.

The different proteoglycans were also examined immunohistochemically in retinal cross-sections (Figure 7). Immunoreactivity of aggrecan was found within the plexiform layers of the retina, although an association with blood vessels was occasionally detectable.

**Figure 8:**
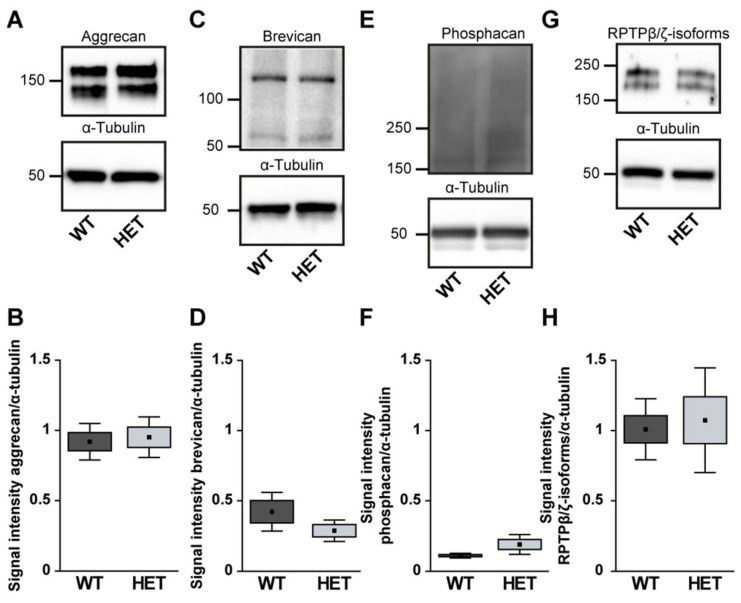
Western blot analyses of relative aggrecan (**A**,**B**), brevican (**C**,**D**), phosphacan (**E**,**F**) and RPTPβ/ζ isoforms (**G**,**H**) in WT and HET retinae. Comparable total protein levels were seen for aggrecan, brevican and all RPTPβ/ζ isoforms. A slightly enhanced signal intensity was detected for phosphacan in the HET condition. Values are indicated as mean ± SEM ± SD. *n* = 4–5/group.

Quantification revealed a comparable immunopositive area for aggrecan in both groups (WT: 17.58 ± 2.82 area [%]/image vs. HET: 14.18 ± 4.19 area [%]/image; *p* = 0.17; Figure 7A–C). Western blot analyses also showed comparable aggrecan protein levels in the WT and HET retina (WT: 0.92 ± 0.13 a.u. vs. HET: 0.95 ± 0.14 a.u.; *p* = 0.76; Figure 8A, B). Brevican staining was found in the GCL and plexiform layers. Our analyses showed a comparable brevican^+^ area in WT (17.82 ± 4.02 area [%]/image) and HET (20.30 ± 6.06 area [%]/image) retinae (*p* = 0.47; Figure 7D–F). Western blotting also demonstrated comparable brevican protein levels in the WT and HET retina (WT: 0.42 ± 0.14 a.u. vs. HET: 0.29 ± 0.08 a.u.; *p* = 0.21; Figure 8C,D).

Phosphacan, which carries the 473HD epitope, represents the secreted splice variant of RPTPβ/ζ (Figure 7G,H). As previously shown, phosphacan staining was restricted to Müller glia and the ILM of the adult retina [9,33]. Interestingly, phosphacan immunoreactivity was significantly higher in the HET (14.30 ± 6.64 area [%]/image) compared to the WT retina (9.15 ± 2.37 area [%]/image; *p* = 0.008; Figure 7I). However, our Western blot analyses showed that protein levels of phosphacan were only in tendency increased in the HET retina (HET: 0.19 ± 0.07 a.u. vs. WT: 0.11 ± 0.02 a.u.; *p* = 0.07; Figure 8E,F). Additionally, comparable levels of all RPTPβ/ζ isoforms were noted in both groups (WT: 17.37 ± 5.06 area [%]/image vs. HET: 13.26 ± 1.73 area [%]/image; *p* = 0.12; Figure 7J–L). RPTPβ/ζ protein bands were detected between 150 and 250 kDa. Here, relative quantification showed comparable protein levels in WT (1.01 ± 0.22 a.u.) and HET (1.07 ± 0.37 a.u.; *p* = 0.75; Figure 8G,H).

### 3.4. Remodeling of Proteoglycans in the Glaucomatous Optic Nerve of PTP-Meg2 HET Mice

**Figure 9:**
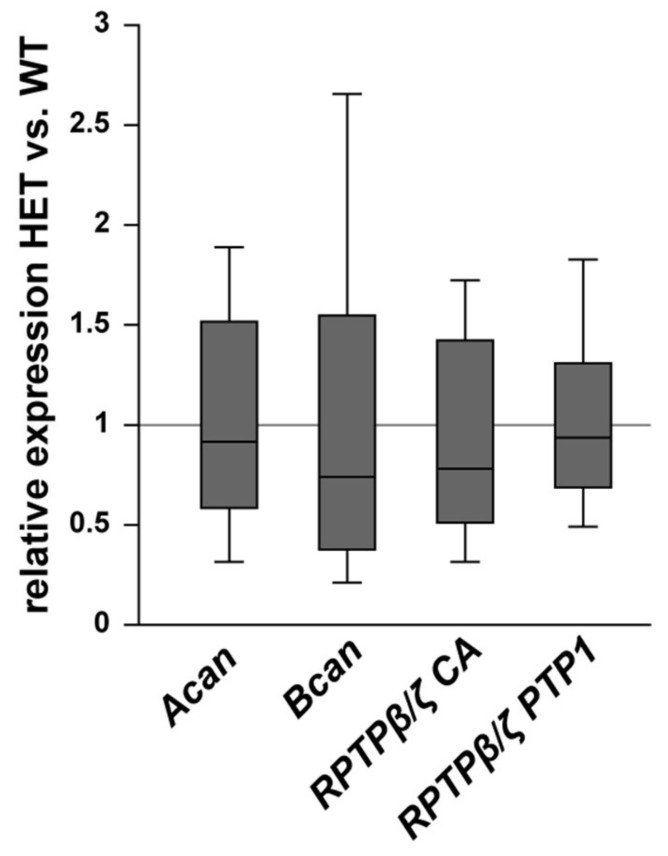
Expression of *Acan*, *Bcan,* and *RPTPβ/ζ* splice variants was comparable in WT and glaucomatous HET optic nerves. On mRNA level, no differences were observed for *Acan*, *Bcan,* and the *RPTPβ/ζ* splice variants detected by the primers *RPTPβ/ζ* CA (all isoforms) and *RPTPβ/ζ* PTP1 (receptor isoforms RPTPβ/ζ_long_ and RPTPβ/ζ_short_). Groups were compared using a pairwise fixed reallocation and randomization test. Data are shown as median ± quartile ± minimum/maximum. *n* = 5/group.

**Figure 10:**
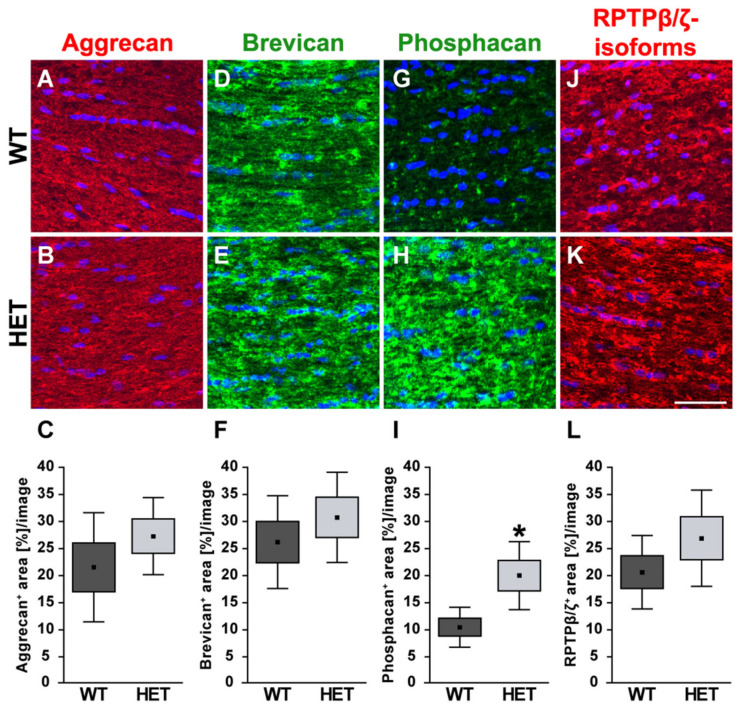
Increased signal area of the 473HD epitope/phosphacan in the glaucomatous optic nerve. Most representative immunohistochemical ECM stainings are shown. Aggrecan (green; (**A**,**B**)), brevican (green; (**D**,**E**)), phosphacan (green; (**G**,**H**)) as well as all RPTPβ/ζ isoforms (red; (**J**,**K**)) showed an immunostaining throughout the optic nerve tissue. Detection of cell nuclei by TO-PRO-3 (blue). Quantification revealed a significant increase in the phosphacan^+^ area in the glaucomatous HET optic nerve (**I**). Comparable signals for all RPTPβ/ζ isoforms (**L**), aggrecan (**C**) and brevican (**F**) were found in both genotypes. Groups were analyzed by Student‘s *t*-test. Data are shown as mean ± SEM ± SD. * *p* < 0.05. *n* = 5/group. Scale bar = 50 μm.

Finally, the staining pattern of the proteoglycans was also characterized in the optic nerve of glaucomatous HET and WT animals. On mRNA level no changes could be found for the analyzed proteoglycans (Figure 9). Here, expression of *Acan* (0.91-fold; *p* = 0.71) and *Bcan* (0.73-fold; *p* = 0.41) was comparable in HET and WT tissue. Additionally, RPTPβ/ζ CA (0.77-fold; *p* = 0.34) as well as the RPTPβ/ζ PTP1 (0.92-fold; *p* = 0.67) expression levels were comparable in both groups. A regular, evenly distributed staining pattern of proteoglycans was seen in the optic nerve (Figure 10). The aggrecan^+^ area was statistically comparable in WT (21.54 ± 10.09 area [%]/image) and HET mice (27.30 ± 7.13 area [%]/image; p = 0.33; Figure 10A–C). Investigation of brevican immunoreactivity also demonstrated unchanged protein level in WT (25.99 ± 8.57 area [%]/image) and HET (30.56 ± 8.33 area [%]/image; *p* = 0.42; Figure 10D–F). In the WT condition a dot-like immunostaining was evident, while a diffuse distribution of phosphacan was noted in the HET group (Figure 10G,H). A doubling of the staining area was observed for the 473HD epitope in HET (19.78 ± 6.29 area [%]/image) compared to the WT (10.27 ± 3.68 area [%]/image; *p* = 0.02; Figure 10I). Interestingly, the assessment of all RPTPβ/ζ isoforms revealed no changes of the immunoreactivity between both genotypes (WT: 20.44 ± 6.78 area [%]/image vs. HET: 26.71 ± 8.89 area [%]/image; *p* = 0.25; Figure 10J–L).

## 4. Discussion

In the present study, we investigated the expression pattern of various ECM molecules in the retina and optic nerve of WT and glaucomatous PTP-Meg2 HET mice via RT-qPCR, immunohistochemistry, and Western blot analyses. PTP-Meg2 HET mice exhibit age-related IOP elevation, which peaks at 28 weeks of age [3]. Therefore, we performed our experiments at 28 weeks of age in WT and HET littermates, when IOP was chronically increased in the glaucoma model.

### 4.1. Increased Levels of the ECM Glycoprotein Fibronectin in the Glaucomatous Retina and Optic Nerve

First, investigations of the glycoproteins fibronectin, laminin, tenascin-C, and tenascin-R were performed in retina and optic nerve tissue. Our results demonstrated significantly enhanced levels of fibronectin in the retina and optic nerve of PTP-Meg2 HET mice. The immunohistochemical analyses revealed that fibronectin was closely associated with blood vessels. Indeed, fibronectin is a key component of the vascular basement membrane of retinal vessels and facilitates cell attachment via integrins [18,52]. Vecino et al. proposed that expression of the receptor α5β1 integrin by RGCs is associated with fibronectin in vitro [53]. Additionally, as a key adhesion protein, fibronectin can interact with various growth factors [54,55,56]. Martino and colleagues demonstrated that the fibronectin binding domain III12-14 has a high binding affinity to the vascular endothelial growth factor (VEGF)-A, an important modulator of neovascularization [57]. Neovascular glaucoma causes the development of abnormal new blood vessels that prevent aqueous humor outflow, which ultimately leads to an increased IOP. Regression of neovascularization by treatment with anti-VEGF agents reduced IOP and led to a better outcome for neovascular glaucoma [58,59]. In this regard, our findings might point to neovascularization in glaucomatous PTP-Meg2 HET mice. Furthermore, enhanced fibronectin levels seem to be associated with chronic IOP elevation, which may be induced by an increased *Vegf* expression.

### 4.2. Altered Isoform-Specific Regulation of Laminin in Glaucomatous Tissue

Laminins are heterotrimers that consist of three disulfide-linked polypeptides, namely α, β, and γ subunits. There are numerous isoforms of each subunit, thus many laminin trimers can be generated [25,60]. For total laminin, we found a significantly increased immunoreactivity in retinal and optic nerve sections of glaucomatous HET mice. In the retina, we observed laminin signals in the ILM and retinal blood vessels. In this regard, Libby et al. described a distinct staining for the laminin α4 chain in close association with Müller glia endfeet as well as in the GCL [22]. In the present study, we also found laminin^+^ cells in the INL. Based on this finding, Li and colleagues verified that amacrine cells express γ3 laminin [61]. We specifically analyzed the mRNA expression level of the laminin isoforms *α4* (*Lama4*), *β2* (*Lamb2*), and *γ3* (*Lamc3*) via RT-qPCR. In retinal tissue, we showed an upregulation of *Lamc3*, while the other isoforms were unchanged in PTP-Meg2 deficient compared to WT mice. Taken together, these findings suggest** that *Lamc3* is upregulated by amacrine cells after glaucomatous damage. However, future analyses should confirm this upregulation on protein level with an antibody directed against laminin γ3.

In contrast to the immunohistochemical analyses, which showed an upregulation of laminin in the HET retina, our Western blot experiments revealed comparable protein levels. Overall, such discrepancies can probably be explained by the different experimental conditions and evaluation strategies. Consequently, variances in the antibody sensitivity, or the analyses of fixed retinal sections and whole denatured retinal tissue, can lead to different results. Moreover, due to the fact that ECM proteins are heavily glycosylated, it is possible that the experimental conditions cause changes in the glycosylation pattern and an altered signal detection.

A reduced expression of *Lama4* and *Lamb2* was found in HET optic nerve tissue, while an unchanged *Lamc3* expression was observed. The laminin α4 chain regulates vascular branching by activating the Delta-like4/Notch signaling cascade in vivo [52]. Laminins that contain the β2 and γ3 chains are essential for migration of astrocytes and blood vessel formation in the retina [27]. Moreover, laminin is important for axonal growth and survival of RGCs in vitro [53,62]. Indeed, a decreased laminin expression is associated with RGC apoptosis in an IOP-dependent model of glaucoma and after optic nerve ligation [63,64]. In our study, glaucomatous PTP-Meg2 HET mice showed a significant reduction in the laminin isoforms *α4* and *β2* in the optic nerve, suggesting that the laminin isoform-specific degradation negatively affect the survival of RGC axons. In summary, our study demonstrated a regulation of specific laminin isoforms in HET mice, indicating that laminin isoforms have distinct functions during glaucomatous degeneration.

### 4.3. Upregulation of Tenascin-C in the Glaucomatous Retina and Optic Nerve

In the adult, the glycoprotein tenascin-C reappears during neurodegenerative processes and is highly associated with glial reactivity and glial scar formation [28,30,40]. During progressive age-related IOP elevation, PTP-Meg2 HET mice show astrogliosis, which is characterized by an increased immunoreactivity of the glial fibrillary acidic protein (GFAP) [3]. We found a significant upregulation of tenascin-C in the retina and optic nerve of glaucomatous PTP-Meg2 HET mice. Studies have shown that tenascin-C immunoreactivity in the optic nerve is limited to astrocytes [9,28,29]. In an experimental rat model, it could be shown that an IOP-induced deformation of the optic nerve head led to an upregulation of various ECM molecules, such as tenascin-C and fibronectin [34]. Furthermore, reactive astrocytes, which express increased tenascin-C levels were found in the optic nerve head of POAG patients [65]. In agreement with the studies described, we show an IOP-dependent change of tenascin-C in the retina and optic nerve in our glaucoma mouse model.

Neuroinflammation plays a key role in glaucomatous degeneration [66]. Tenascin-C plays an immunomodulatory role in neuroinflammatory as well as autoimmune diseases [30,67]. Midwood et al. showed that tenascin-C supports pro-inflammatory processes via TLR4 signaling [68]. In addition, a recent study on primary microglia has shown that tenascin-C stimulates the release of pro-inflammatory tumor necrosis factor alpha and induces the expression of the inducible nitric oxide synthetase by the TLR4 pathway [69]. Accordingly, alterations in ECM components affect the activation, differentiation, and migration of microglia. In a previous study, we confirmed an increased microglia reactivity upon IOP elevation in glaucomatous PTP-Meg2 HET retina and optic nerve [3]. It is therefore tempting to speculate that tenascin-C-mediated signaling plays an important role in the activation and migration of astrocytes and microglia, which has a harmful effect on RGC survival in our glaucoma mouse model. Consistent with this assumption, we recently demonstrated that *Tnc* knock-out mice exhibit less severe IOP-independent glaucomatous damage of RGCs and optic nerve fibers, decreased micro- and macroglial reactivity, and increased anti-inflammatory cytokine expression [70].

### 4.4. Unchanged levels of Tenascin-R

The glycoprotein tenascin-R is predominantly expressed by optic nerve oligodendrocytes and horizontal cells of the retina [28,38,39]. Tenascin-R can influence neurite outgrowth as well as neural and glial adhesion [35,36,37]. A domain-specific neuroprotective effect of tenascin-R has been described, presumably by regulation of microglia behavior [71]. It has also been speculated that an isoform-specific regulation of tenascin-R occurs under ischemic conditions [16]. However, our experiments showed no alteration in tenascin-R protein level or mRNA expression in PTP-Meg2 HET mice. An unchanged protein level of tenascin-R was also reported in an optic nerve crush model [72]. Based on the unaltered tenascin-R levels upon IOP-elevation, tenascin-R expressing cells in our glaucoma model do not seem to respond to damage.

### 4.5. Increased Expression of the RPTPβ/ζ isoform Phosphacan in the Glaucomatous Retina

Investigations of PTP-Meg2 deficient mice showed astrogliosis and glial scar formation as a reaction to glaucomatous RGC damage after IOP increase. An accumulation of CSPGs in the glial scar limits the regeneration of neuronal tissue [73,74]. Here, we analyzed the expression pattern of the CSPGs aggrecan, brevican, and phosphacan as well as the RPTPβ/ζ receptor isoforms RPTPβ/ζ_long_ and RPTPβ/ζ_short_.

An increased accumulation of aggrecan and brevican near the lesion core was recognizable after optic nerve crush in rats [44]. An upregulation of both proteoglycans has also been described under ischemic conditions [16]. In hereditary retinal dystrophies, however, aggrecan is not involved in degenerative processes [75]. Remarkably, we found comparable protein and mRNA levels of these proteoglycans, which indicates a damage-independent expression of aggrecan and brevican in our glaucoma model.

Tenascin-C is a high-affinity ECM ligand of RPTPβ/ζ/phosphacan [76,77]. Our RTq-PCR and immunohistochemical results demonstrated a significant upregulation of the secreted RPTPβ/ζ isoform phosphacan. Additionally, our Western blot analyses showed a trend towards higher phosphacan protein levels. Statistical analyses, however, did not reveal significant differences (*p* = 0.07), which could possibly be explained by the sensitivity of the different evaluation strategies as well as the existing experimental conditions. In the retina, phosphacan was continuously and exclusively expressed by Müller glia in WT and HET mice. A Müller glia specific expression of phosphacan has been described in vitro and in vivo [9,45].

Analyses of the RPTPβ/ζ receptor variants showed no differences in both genotypes. An enhanced phosphacan staining signal restricted to Müller glia as well as to optic nerve astrocytes, has also been described in an IOP-independent autoimmune glaucoma rat model [33]. Macrogliosis was shown not only by an increased number of GFAP-positive astrocytes, but also by an increased vimentin staining of Müller glia in HET mice [3]. As shown for tenascin-C, the phosphacan signal area in the optic nerve was associated with glial cells. Due to the specific phosphacan immunoreactivity in Müller glia and astrocytes, we assume that accumulation of RPTPβ/ζ/phosphacan and its interaction partner tenascin-C is directly related to reactive gliosis in glaucomatous HET mice.

### 4.6. Impact of ECM Changes on Glaucomatous RGC Death

Our study provides evidence of ECM remodeling in the optic nerve and retina after IOP-induced glaucomatous damage. We speculate that alterations of the ECM may have a direct impact on RGC death and optic nerve degeneration by influencing survival and apoptotic signaling pathways. Previous studies suggest that abnormal ECM remodeling in the glaucomatous retina is related to the death of RGC [63,64,78]. The ECM also affects integrin expression of RGCs, thus influencing their survival and axonal growth [53]. It has also been shown that the ECM is capable to bind, sequester and locally release various growth factors [79]. In this regard, it could be possible that ECM stiffness has an influence on the accessibility of specific growth factor receptors and therefore affects the survival of RGCs. However, the ECM is a multitasking player, which can also have an influence on RGCs through the orchestration of micro- and macroglial cells. Nevertheless, further studies are necessary to identify the molecular signaling pathways underlying the ECM changes in glaucomatous RGC death. This knowledge could also be useful in advancing the development of matrisome technology and potent pharmacotherapies for the treatment of retinal and optic nerve injury [80,81].

## 5. Conclusions

In this study, we demonstrated ECM remodeling in the IOP-dependent PTP-Meg2 HET glaucoma model. The upregulation of the blood vessel-associated ECM component fibronectin indicates neovascularization during glaucomatous damage. The expression pattern of different laminin subunits suggests an isoform-specific regulation. The expression pattern and increased levels of tenascin-C and phosphacan might point to their functional involvement in glaucomatous gliosis. Furthermore, dysregulation of ECM molecules seems to be rather harmful than neuroprotective. Future studies should focus on ECM signaling pathways for a better understanding of the underlying molecular mechanisms of RGC death and gliotic activity in IOP-dependent glaucomatous damage. This overall knowledge could be important for the development of new and improved diag-nostic and therapeutic approaches for patients with glaucoma.

## Figures and Tables

**Table 1:** List of primer pairs that were used for mRNA analyses in WT and HET retinae and optic nerves via RT-qPCR. To normalize the ECM expression data, the expression of the housekeeping genes *β-actin* (*Actb*) and *cyclophilin D* (*Ppid*) was assessed. The table shows the primer names, gene names, primer sequences, GenBank accession numbers, and product sizes. Abbreviations: bp—base pairs, for—forward, rev—reverse.

Primer Name	Gene Name	Primer Sequence	GenBank Accession Number	Product Size (bp)
*β-actin*_for	*Actb*	ctaaggccaaccgtgaaaag	NM_007393.5	104
*β-actin_*rev		accagaggcatacagggaca		
*Aggrecan*_for	*Acan*	ccagcctacaccccagtg	NM_007424.3	66
*Aggrecan*_rev		gagggtgggaagccatgt		
*Brevican*_for	*Bcan*	tcaatgtctactgcttccgaga	NM_007529.2	72
*Brevican_*rev		catctgaggctgggctagag		
*Cyclophilin D*_for	*Ppid*	aaggatggcaaggattgaaa	NM_026352.4	105
*Cyclophilin D*_rev		ctttaagcaattctgcctgga		
*Fibronectin*_for	*Fn1*	gccaccggagtctttactacc	NM_010233.2	61
*Fibronectin_*rev		cctcggtgttgtaaggtgga		
*Laminin α4*_for	*Lama4*	tgtttgttggaggtgttcca	NM_010681.4	130
*Laminin α4_rev*		ctgaccagggcagctttact		
*Laminin* *β2* *_for*	*Lamb2*	tttcatttcacccacctcatt	NM_008483.3	77
*Laminin* *β2* *_rev*		aagtctgcagaacgctccac		
*Laminin γ* *3* *_for*	*Lamc3*	gctgacctcagaagcacaca	NM_011836.4	70
*Laminin γ* *3* *_rev*		cacagtgctcagcccaga		
*RPTPβ/ζ* CA_for	*RPTPβ/ζ*	gaatcctgcagagcttcctc	NM_011219.2	74
*RPTPβ/ζ* CA_rev		gtagtatccataagcccagtcca		
*RPTPβ/ζ* PTP1_for		ttgacggttccttcatgttg	NM_001311064.1	101
*RPTPβ/ζ* PTP1_rev		ttgacggttccttcatgttg		
*Tenascin-C*_for	*Tnc*	cagggatagactgctctgagg	NM_001369211.1	90
*Tenascin-C_*rev		cattgtcccatgccagattt		
*Tenascin-R*_for	*Tnr*	gatggaagccgcaaagag	NM_022312.3	68
*Tenascin-R*_rev		tctgacaggccctctagtcg		

**Table 2:** Primary and secondary antibodies for immunohistochemistry.

Primary Antibody	Dilution	Reference/Source	Secondary Antibody	Dilution	Source
Aggrecan	1:250	Millipore	Goat anti-rabbit Cy3	1:250	Jackson ImmunoResearch Labs
Brevican	1:300	[46]	Goat anti-guinea pig Cy2	1:250	Jackson ImmunoResearch Labs
Fibronectin	1:300	[47]	Goat anti-rabbit Cy3	1:250	Jackson ImmunoResearch Labs
Laminin	1:300	[47]	Goat anti-rabbit Cy2	1:250	Jackson ImmunoResearch Labs
Tenascin-C (KAF14 antibody)	1:250	[48]	Goat anti-rabbit Cy3	1:250	Jackson ImmunoResearch Labs
Tenascin-R (23-14 antibody)	1:100	[49]	Goat anti-mouse Cy2	1:250	Jackson ImmunoResearch Labs
Phosphacan/RPTPβ/ζ (473HD antibody)	1:200	[50]	Goat anti-rat Cy2	1:250	Jackson ImmunoResearch Labs
RPTPβ/ζ-isoforms (KAF13 antibody)	1:200	[50]	Goat anti-rabbit Cy3	1:250	Jackson ImmunoResearch Labs

**Table 3:** Adjustments for the ImageJ macro. Background subtraction data and the upper and lower threshold values for determining the stained signal area [%]/image.

Protein	Tissue	Background Subtraction	Lower Threshold	Upper Threshold
Aggrecan	Retina	50	7.55	78.88
	Optic nerve	50	13.84	80.10
Brevican	Retina	20	5.21	35.24
	Optic nerve	20	12.18	72.30
Fibronectin	Retina	50	23.45	78.31
	Optic nerve	50	10.90	78.85
Laminin	Retina	30	11.25	75.65
	Optic nerve	30	33.98	79.83
Tenascin-C	Retina	50	25.33	80.11
	Optic nerve	50	18.50	73.50
Tenascin-R	Retina	20	20.00	80.00
	Optic nerve	50	21.22	79.99
RPTPβ/ζ (473HD)	Retina	50	8.77	79.22
	Optic nerve	20	18.80	75.30
RPTPβ/ζ (KAF13)	Retina	50	18.01	77.02
	Optic nerve	50	22.53	80.00

**Table 4:** Primary and secondary antibodies for Western blotting.

Primary Antibody	Molecular Weight	Dilution	Reference/Source	Secondary Antibody	Dilution	Source
Aggrecan	>100 kDa, >150 kDa	1:1000	Millipore	Goat anti-rabbit HRP	1:10,000	Jackson ImmunoResearch Labs
Brevican	~50 kDa, >100 kDa	1:1000	[46]	Goat anti-guinea pig HRP	1:5000	Jackson ImmunoResearch Labs
Fibronectin	>250 kDa	1:10,000	[47]	Goat anti-rabbit HRP	1:10,000	Jackson ImmunoResearch Labs
Laminin	200 kDa, 400 kDa	1:10,000	[47]	Goat anti-rabbit HRP	1:5000	Jackson ImmunoResearch Labs
Tenascin-C (KAF14 antibody)	~250 kDa, >250 kDa	1:5000	[48]	Goat anti-rabbit HRP	1:10,000	Jackson ImmunoResearch Labs
Tenascin-R (23–14 antibody)	160 kDa, 180 kDa	1:1000	[49]	Goat anti-mouse HRP	1:5000	Jackson ImmunoResearch Labs
Phosphacan/RPTPβ/ζ (473HD antibody)	>150 kDa	1:100	[50]	Goat anti-rat HRP	1:5000	Jackson ImmunoResearch Labs
RPTPβ/ζ-isoforms (KAF13 antibody)	>150 kDa	1:5000	[50]	Goat anti-rabbit HRP	1:5000	Jackson ImmunoResearch Labs
α-Tubulin	~50 kDa	1:20,000	Sigma-Aldrich	Goat anti-mouse HRP	1:10,000	Jackson ImmunoResearch Labs

## Data Availability

The data presented in this study are available on request from the corresponding author.

## Abbreviations

Bp	base pairs
CA	carbonic anhydrase-like domain
CSPG	chondroitin sulfate proteoglycan
ECM	extracellular matrix
For	forward
GCL	ganglion cell layer
GFAP	glial fibrillary acidic protein
HET	heterozygous
ILM	inner limiting membrane
INL	inner nuclear layer
IOP	intraocular pressure
IPL	inner plexiform layer
ONL	outer nuclear layer
OPL	outer plexiform layer
PBS	phosphate-buffered saline
POAG	primary open-angle glaucoma
PTP1	protein tyrosine phosphatase domain 1
PTP-Meg2	protein tyrosine phosphatase megakaryocyte 2
Rev	reverse
RGC	retinal ganglion cell
RPTPβ/ζ	receptor protein tyrosine phosphatase beta/zeta
RT-qPCR	quantitative real-time polymerase chain reaction
SD	standard deviation
SEM	standard error of the mean
TLR4	toll-like receptor 4
WT	wild type
